# Codon usage bias and environmental adaptation in microbial organisms

**DOI:** 10.1007/s00438-021-01771-4

**Published:** 2021-04-05

**Authors:** Davide Arella, Maddalena Dilucca, Andrea Giansanti

**Affiliations:** 1 Department of Physics, Sapienza University of Rome, 00185 Rome, Italy; 2 Department of Physics, Sapienza University of Rome, 001885 Rome, Italy; 3 INFN, Roma1 Unit, 00185 Rome, Italy

**Keywords:** Codon bias, Environment, Adaptation, tRNA, Bacteria, Archaea

## Abstract

In each genome, synonymous codons are used with different frequencies; this general phenomenon is known as codon usage bias. It has been previously recognised that codon usage bias could affect the cellular fitness and might be associated with the ecology of microbial organisms. In this exploratory study, we investigated the relationship between codon usage bias, lifestyles (thermophiles vs. mesophiles; pathogenic vs. non-pathogenic; halophilic vs. non-halophilic; aerobic vs. anaerobic and facultative) and habitats (aquatic, terrestrial, host-associated, specialised, multiple) of 615 microbial organisms (544 bacteria and 71 archaea). Principal component analysis revealed that species with given phenotypic traits and living in similar environmental conditions have similar codon preferences, as represented by the relative synonymous codon usage (RSCU) index, and similar spectra of tRNA availability, as gauged by the tRNA gene copy number (tGCN). Moreover, by measuring the average tRNA adaptation index (tAI) for each genome, an index that can be associated with translational efficiency, we observed that organisms able to live in multiple habitats, including facultative organisms, mesophiles and pathogenic bacteria, are characterised by a reduced translational efficiency, consistently with their need to adapt to different environments. Our results show that synonymous codon choices might be under strong translational selection, which modulates the choice of the codons to differently match tRNA availability, depending on the organism’s lifestyle needs. To our knowledge, this is the first large-scale study that examines the role of codon bias and translational efficiency in the adaptation of microbial organisms to the environment in which they live.

**Supplementary Information:**

The online version supplementary material available at 10.1007/s00438-021-01771-4.

## Introduction

Following a quite shared view, microbial evolution is mainly driven by the adaptation to the environment (see, e.g. the nice database paper from Yana Bromberg’s lab (Zhu et al. 2018). Then, organisms living in the same ecological niche should have evolved similar functional traits, different from those of the species that thrive in different environments. In this paper, we explore whether microbes living in similar environments and sharing similar lifestyles also share, at the genetic level, similar signatures of codon bias. As is well known, the genetic code is degenerate. Most of the amino acids are encoded by more than one codon. Although coding for the same amino acid, synonymous codons are not equally frequent, a phenomenon known as codon usage bias (CUB) (Grantham et al. 1980). Codon usage might differ widely not only between organisms, but also within a genome and within a single gene (Hooper and Berg 2000; Plotkin and Kudla 2011). A lot of factors might cause different CUB and the selective forces influencing it, such as selection for optimised translation, expression and location in genes, rate of evolution, secondary structure, nucleotide composition, protein length and environment (Salim and Cavalcanti 2008). It was demonstrated that many bacteria and yeast undergo translational selection, with highly expressed genes preferentially using codons that are translated faster and/or more accurately by the ribosome (Gouy and Gautier 1982; Bennetzen and Hall 1982). Thus, the CUB within a genome usually reflects the selection pressure for translational optimisation of highly expressed genes. The choice of preferred codons in a single genome is closely correlated with abundance of the cognate tRNA molecules (Bennetzen and Hall 1982; Ikemura 1981, 1985; Dong et al. 1996) and further influenced by the genome’s GC content (Chen et al. 2004; Hershberg and Petrov 2009). It was argued by Andersson and Kurland (1990) and then substantiated by Kudla and his group (Kudla et al. 2009) that selection towards highly adapted codons in highly expressed proteins has a global effect on the cell, resulting in an increased cellular fitness. This suggests that the global extent of CUB of an organism might be associated with its phenotypic traits. Following this idea, Botzman and Margalit determined an association between the lifestyles of several microbial organisms and variations in their codon usage (Botzman and Margalit 2011). Their results indicated that species living in a wide range of habitats have low CUB, which is consistent with the need to adapt to different environments. Furthermore, it has been demonstrated that microbes living in the same ecological niche share a common preference for codon usage, regardless of their phylogenetic diversity (Roller et al. 2013). Complementing these studies, the analysis of acidophilic bacteria revealed that they preferentially have a low codon bias (all the codons tend to be equally frequent), that is compatible with their capacity to live in a wide range of habitats (Hart et al. 2018). Overall, the above quoted literature suggested the present investigation in which we explore whether lifestyles and habitats of microbial species are correlated with their codon bias, tRNA availability and an index (tAI) that can be reasonably associated to their efficiency in producing proteins. We have considered 615 species, 544 bacterial and 71 archaeal. We used the relative synonymous codon usage (RSCU) index as a statistic useful to characterise the CUB of single genes and of entire genomes. We then considered tRNA availability and tRNA adaptation index (tAI) to study the integration between CUB and tRNAs as a fundamental adaptive trait of an organism to its environment. In the next sections, we show that organisms sharing specific phenotypic characteristic (lifestyles) and living in similar environmental conditions (habitats) have similar codon usage features, a similar tRNA availability spectrum and similar distributions of tAI. We used principal component analysis (PCA) and basic statistical tests on tAI distributions. Our observations reinforce the interpretation of the codon bias that emerges from the evolution of a microbial species as the result of multiple trade-offs between translation efficiency, biosynthetic costs, and varied availability of nutrients typical of different environments.

## Materials and methods

### Dataset of bacterial and archaeal species

We analyzed the CUB in the genomes of 615 microbial organisms—544 bacteria and 71 archaea—reported in Online Resource 1 (see Supplementary Table 1). The dataset comprised a wide range of bacterial families. We have included also a set of Archaea to search for robust signatures of codon bias adaptation to different environments and lifestyles, that could emerge in microbial species beyond the distinction of superkingdom. The environmental characteristics of the species that we considered (temperature range, oxygen requirement, salinity, habitat and pathogenicity) were downloaded from the Additional file 2 of the paper by Botzman and Margalit (2011). The nucleotide sequences of the protein coding regions, also known as CDSs (from coding sequences) of the genome of each species were downloaded from the GenBank database (Benson et al. 2012) using the FTP site of the National Center for Biotechnology Information (ftp://ftp.ncbi.nlm.nih.gov). The tRNA gene copy number (tGCN) for each organism was retrieved from the Genomic tRNA database (GtRNAdb) (Lowe and Eddy 1997) available at the site http://gtrnadb.ucsc.edu. In Table 1 we show the class of the species we have considered. In Table 2 we make a summary of the number of species in our dataset, classified according their ecological adaptation and lifestyle. Overall, we believe to have sufficiently sampled both microbial phylogeny and ecological adaptation.

**Table 1:** Taxonomy of the microbial organisms in the dataset

Superkingdom	Taxonomy (class)	Number of genomes
ARCHAEA	Archaeoglobi	3
	Methanobacteria	3
	Methanococci	12
	Methanomicrobia	10
	Methanopyrales	2
	Halobacteria	10
	Nanoarchaeales	2
	Thermococci	6
	Thermoplasmata	3
	Thermoprotei	20
BACTERIA	Acidobacteria	1
	Actinobacteria	39
	Alphaproteobacteria	69
	Aquificae	4
	Bacteroides	15
	Betaproteobacteria	46
	Bdellovibrionales	1
	Chlamydiae	8
	Chlorobi	3
	Chloroflexi	4
	Clostridia	3
	Cyanobacteria	32
	Deinococci	4
	Deltaproteobacteria	16
	Epsilonproteobacteria	17
	Firmicutes	111
	Fusobacteria	1
	Gammaproteobacteria	136
	Heunggongvirae (virus)	1
	Mollicutes	18
	Opitutae	1
	Spirochaete	9
	Thermotogae	5

**Table 2:** Classification of the microbial organisms in the dataset by phenotypic traits

Phenotypic trait	Number of species
Temperature range	
Hyperthermophilic	37
Thermophilic	39
Mesophilic	490
Psychrophilic	9
Pathogenicity	
Pathogenic	276
Non-pathogenic	257
Oxygen requirement	
Aerobic	196
Anaerobic	117
Facultative	207
Microaerophilic	18
Salinity	
Extreme halophilic	9
Moderate halophilic	14
Mesophilic	19
Non-halophilic	112
Habitat	
Aquatic	119
Terrestrial	36
Host-associated	211
Specialised	72
Multiple	177

### RSCU calculation

The relative synonymous codon usage (RSCU) (Sharp et al. 1986) is the observed frequency of a codon divided by the expected frequency if all the synonymous codons were used equally. The RSCU is computed for each codon of each amino acid and it is formally defined as follows. Let $n_i$ denote the number of synonymous codons encoding for the amino acid *i* (codon degeneracy) and let $X_{ij}$ denote the number of occurrences of the codon *j* for amino acid *i*. The RSCU for codon *j* encoding the amino acid *i* is defined as

1 $$\begin{aligned} \text {RSCU}_{ij} = \frac{X_{ij}}{\frac{1}{n_i}\sum \nolimits _{j=1}^{n_i} X_{ij}} \, . \end{aligned}$$

RSCU is a real value comprised between 0 and the number of synonymous codons for that amino acid, i.e. $n_i$ . For average synonymous codon usage (no codon bias), the RSCU is 1. For codon usage more infrequent than the average codon usage, the RSCU is less than one, and for more frequent usage than the average for the amino acid, the RSCU is greater than 1.

We calculated the RSCU values of each codon for all CDSs (genes) of the different organisms.The RSCU values of the various codons can be considered as the 61 components (excluding the stop codons TAA, TAG and TGA—which are differently used by different species) of vectors which measure CUB in a given gene.

For each genome, we calculated the average vector of RSCU, $\overline{\text{ RSCU }}$, and the similarity between the RSCU vector of a gene and the $\overline{\text{ RSCU }}$ vector of the genome. As a measure of similarity between two vectors, we used the cosine similarity from the following formula:

2 $$\begin{aligned} \text {similarity} = \text {cos}\theta = \frac{\text {RSCU}\bullet {\overline{\mathrm{RSCU}}}}{\parallel \text {RSCU}\parallel \,\parallel {\overline{\mathrm{RSCU}}}\parallel }\, , \end{aligned}$$

where $\bullet$ denotes the scalar product and $\parallel \text {RSCU}\parallel$ is the magnitude of the RSCU vector. When the cosine similarity is 1 the two vectors have the same orientation, whereas if the cosine similarity is 0 they are orthogonal to each other.

A few methodological remarks on the metric of our choice are in order at this point. There are several methods to measure the similarity or the dissimilarity between two vectors. The Euclidean distance is one of the mostly used, due to its simplicity. In the Euclidean space, the distance between two points is measured by the length of the line segment connecting them. Unfortunately, the Euclidean distance suffers from a high sensitivity to the magnitudes of the vectors that are compared (Xia et al. 2015). Cosine similarity is commonly used in information retrieval (Korenius et al. 2007) and data mining (Tan et al. 2016), particularly in high-dimensional positive spaces. We chose cosine similarity because it is normalised by the moduli of the vectors, then, by default, insensitive to magnitudes. Cosine similarity focusses on the relative directions of the vectors that are compared, measuring the cosine of the angle between the vectors. Vectors that convey similar information and meaning have robustly, in this representation, a small angle between them. Vectors that have substantially the same direction (most of the components have similar values) but accidentally differ just in one component my have a relatively large (spurious) Euclidean distance.

All sequence manipulations were carried out using in-house programs written in the Python language (https://www.python.org/) and the figures were drawn with the Python data visualization libraries Matplotlib (https://matplotlib.org/) and Seaborn (https://seaborn.pydata.org/).

Let us also add another methodological remark. In this paper, we did not split into two families the synonymous codons of the amino acids Arginine, Leucine and Serine, with sixfold degeneracy. In particular, in the usual normalization of the RSCU values, we plainly adopted here, all the six codons were treated as equivalent. Splitting sixfold degenerate synonymous codons into two families (as suggested in a classic paper (Akashi 1995)) has found a rationale in many contexts, when dealing with the detailed balance of non synonymous, synonymous and nonsense mutations. This distinction is quite relevant in genetic studies devoted to measuring differential selective pressures on specific groups of control genes, often related to the onset of complex pathologies in multicellular organisms (Chu and Wei 2019). In the context of ecological adaptation of unicellular species, it has been shown, in *E. coli*, that environmental perturbations (such as nutrient deprivation) can lift the degeneracy of the genetic code (Subramaniam et al. 2013). In particular, protein production levels are subtly affected, under starving conditions, by the choice of the codon that codes for the sixfold degenerate amino acids. This observation suggests that in future studies of the codon adaptation to different environments, special care should be given, to check whether all the codons in a sixfold degenerate family are indeed synonymous or not in microbial organisms.

### Principal component analysis

Principal component analysis (PCA) (Hotelling 1933; Jolliffe 2002) is a multivariate statistical method to transform a set of observations of possibly correlated variables into a set of linearly uncorrelated variables (called principal components) spanning a space of lower dimensionality. The transformation is defined so that the first principal component accounts for the largest possible variance of the data, and each succeeding component in turn has the highest variance possible under the constraint that it is orthogonal to (i.e. uncorrelated with) the preceding components.

We used this technique on the space of $\overline{\text{ RSCU }}$ values, where each organism of the dataset was represented as a 61-dimensional vector with the codons as coordinates. The eigenvectors of the associated covariance matrix, ordered according to the magnitude of the corresponding eigenvalues, are the principal components of the original data.

The PCA was performed using the open source software gretl (http://gretl.sourceforge.net). We projected in the plane of the first two principal components all the genomes of the dataset. Centroids were calculated as mean values with relative error bars as standard deviation of the mean.

We then carried out another PCA using the number of tRNA gene copies (tGCN) provided by the GtRNAdb, to consider the availability of tRNA for each organism. In this case, the organisms were represented as vectors in the 61-dimensional space of the anticodons.

### tAI calculation

The speed of protein synthesis is bound to the waiting time for the correct tRNA to enter the ribosomal A site (Varenne et al. 1984), and thus depends on tRNA concentrations (Sørensen et al. 1989). The consequent adaptation of codon usage to tRNA availability (Ikemura 1981, 1985) is at the basis of tRNA adaptation index (tAI) (Reis et al. 2004; Dos Reis et al. 2003). It takes advantage of the fact that the tGCN across many genomes has a high and positive correlation with tRNA abundance within the cell (Ikemura 1981; Percudani et al. 1997; Kanaya et al. 1999; Duret 2000). The tAI follows the same mathematical model of CAI (Sharp and Li 1987)—defining for each codon *i* its absolute adaptiveness ($W_i$):

3 $$\begin{aligned} W_i = \overset{m_i}{\underset{j=1}{\sum }}\left( 1-s_ {ij}\right) \text{ tGCN}_{ij} \, , \end{aligned}$$

where $m_i$ is the number of tRNA isoacceptors that recognise the *i* th codon, tGCN$_{ij}$ is the gene copy number of the *j* th tRNA that recognises the *i*-th codon and $s_{ij}$ is a selective constraint on the efficiency of the codon–anticodon coupling. From the $W_i$ values, the relative adaptiveness value $w_i$ of a codon is obtained as

4 $$\begin{aligned} w_ i = {\left\{ \begin{array}{ll} W_ i/W_ {\text {max}}&{}\text{ if } \, W_i\ne 0\\ w_{\text {mean}}&{}\text{ else } \end{array}\right. } \, , \end{aligned}$$

where $W_{\text {max}}$ is the maximum $W_i$ value and $w_{\text {mean}}$ is the geometric mean of all $w_i$ with $W_i\ne 0$. Finally, the tRNA adaptation index $\text {tAI}_g$ of a gene *g* is computed as the geometric mean of the relative adaptiveness values of its codons

5 $$\begin{aligned} \text {tAI}_g=\left( \, \overset{l_g}{\underset{k=1}{\prod }}w_k \right) ^{1/l_g} \, , \end{aligned}$$

where *k* is the codon defined by the *k* th triplet in gene *g* and $l_g$ is the length of the gene in codons (except the stop codon). The tAI of a coding sequence ranges from 0 to 1, with high values corresponding to high levels of translational efficiency. The critical issue for tAI is the selection of a meaningful set of $s_{ij}$ values, i.e. weights that represent the efficiency of the interactions between codons and tRNAs. Assuming that tRNA usage is maximal for highly expressed genes, these values are chosen to optimise the correlation of tAI values with expression levels. We computed tAI values using the package provided by Mario dos Reis on GitHub (https://github.com/mariodosreis/tai). This is an R package that implements the tAI as described in (Reis et al. 2004). For all the organisms in the dataset, we computed the tAI value of each gene and the average tAI value over all genes in a genome, $\overline{\text{ tAI }}$. In this work we tend to consider tAI as a proxy for translation efficiency (and even for the speed of protein synthesis). However, this correlation, although reasonable, is still quite speculative and definitely deserves further study. A correlation between tAI and ribosomal profiling is the direct way to empirically check the view that we share here. This correlation has been shown in eukaryotic species (see, e.g. figure 1C in Chu and Wei 2020 and figure 2C in Wu et al. 2019). To our knowledge, a database in which ribosomal profiling data are correlated with codon bias indicators such as tAI is still lacking, at least for unicellular organisms. Moreover, high-resolution ribosomal profiling experiments in bacteria have their specific difficulties, as pointed out in (Woolstenhulme et al. 2015).

We divided the organisms into groups according to their environmental characteristics and pathogenicity (see Table 2 above) and then we compared the distributions of the average tAI values of the species belonging to those groups. Mann–Whitney *U* test and Kruskal–Wallis *H* test were used to verify whether the differences between the distributions were statistically significant (ritually, with *p* value < 0.05) or not. Significance tests were implemented in Python using the statistical functions of the SciPy library (https://www.scipy.org/).

## Results

### Distribution of RSCU values: intra- and inter-species

**Fig. 1:**
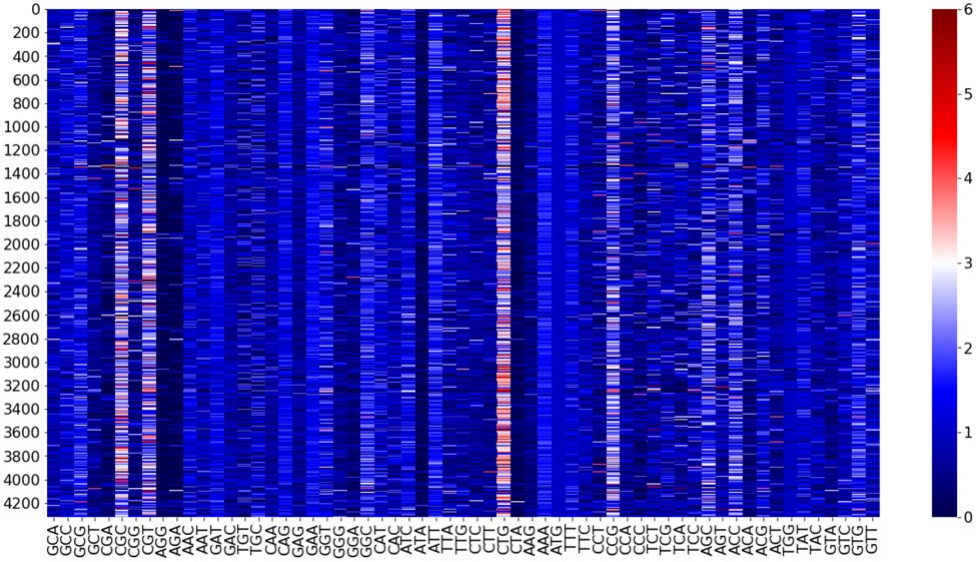
Heat map of RSCU values for each gene of *E. coli* strain K12 substrain MG1655. The 4319 CDSs are given in the rows and the 61 codons are in columns. Codons are shown in the alphabetical order of the amino acids they code for, i.e. from the four synonymous codons encoding Ala to the four ones encoding Val. We note that RSCU vectors of different genes are very similar to each other

**Fig. 2:**
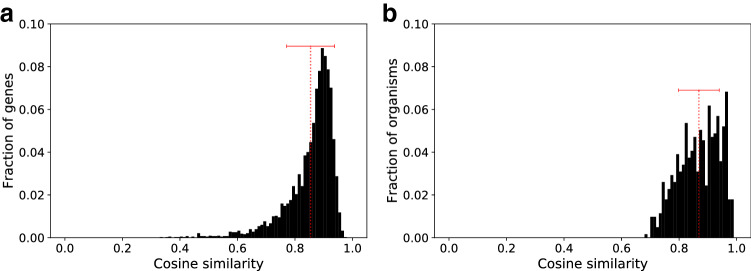
Distribution of cosine similarities. **a** Normalised histogram, taken as an example of intra-species variability of codon bias, of the cosine similarities between the RSCU of individual CDSs (genes) of *E. coli* strain K12 substrain MG1655 and the average vector $\overline{\text{ RSCU }}$. Mean (0.85) and standard deviation (0.08) of the distribution are plotted in red. **b** Normalised histogram of the cosine similarities between the average vectors $\overline{\text{ RSCU }}$ associated to each one of the 615 species in the dataset, and their overall average. This histogram represents the inter-species variability of the codon bias. Red dotted line denotes the mean (0.87) of the distribution and the error bar the standard deviation (0.07)

Previous observations (see Grantham et al. 1980; Bennetzen and Hall 1982; Plotkin and Kudla 2011) pointed to the fact that each bacterial species has a specific pattern of CUB, which is shared by the majority of its genes; codon bias in specialised categories of genes appears to be just a modulation of the distinctive codon bias of the species (Dilucca et al. 2015). To check this view, that is not widely shared, we computed the RSCU values for all the coding regions of each microbe in the dataset. In Fig. 1 is shown, as a representative example, the heat map of RSCU values of all the coding regions (genes) of *E. coli* (K12 substrain MG1655). The map shows that all the genes of the organism share a common pattern of codon bias, that could be assumed as a kind of fingerprint of each species. It is then important to compare the fluctuations in the RSCU of the genes internal to a species with the fluctuations of average RSCU vectors, representative of the codon bias of each species. In Fig.  2 we compared the distribution of the cosine similarities between the RSCU vectors of each gene and the $\overline{\text{ RSCU }}$ vector of *E. coli* (panel a) and the distribution of the similarities between the $\overline{\text{ RSCU }}$ of each species and their overall average vector (panel b). We had in mind to check that the intra-variability is definitely smaller than the inter-variability. A comparison of the numerical values of the averages and the standard deviations in the two cases does not show the striking difference we expected, but the shape of the two distributions is definitely different suggesting that the two distributions correspond to different statistical models that it would be interesting to define using a more extended set of data, in a dedicated study. In the context of the present work, we assume that the wide distribution of inter- species RSCU variability (panel b) justifies the investigation (in the space of the principal components of the average RSCU vectors, as done below) of the correlation between codon bias patterns of each species and their adaptation to the environment.

### Principal component analysis

**Fig. 3:**
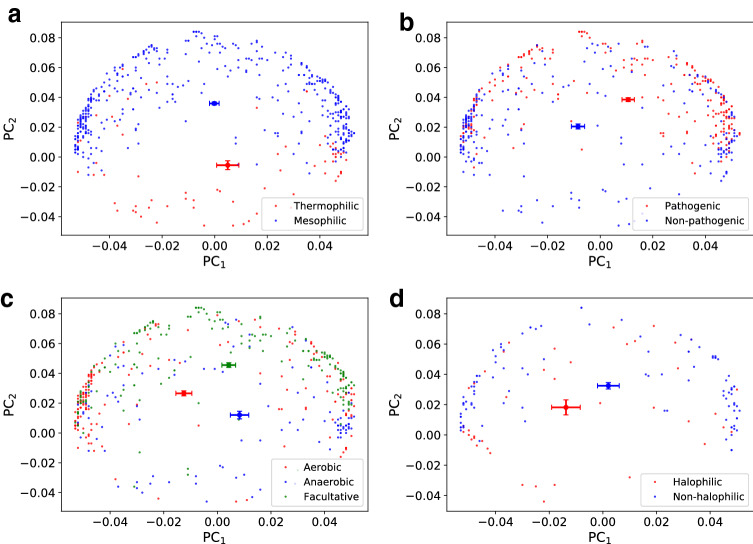
PCA using $\overline{\text{ RSCU }}$ vectors of all the organisms in the dataset. The first principal component (PC$_1$) accounted for 51.6% of the total variation and the second principal component (PC$_2$) accounted for 19.7% of the total variation. We projected in the PC$_1$–PC$_2$ plane the organisms with different colors, according to their phenotypic traits: temperature range (**a**), pathogenicity (**b**), oxygen requirement (**c**), salinity (**d**). Centroids were calculated as mean value with relative error bars as standard deviation of the mean. The centroids of each group are well separated

**Fig. 4:**
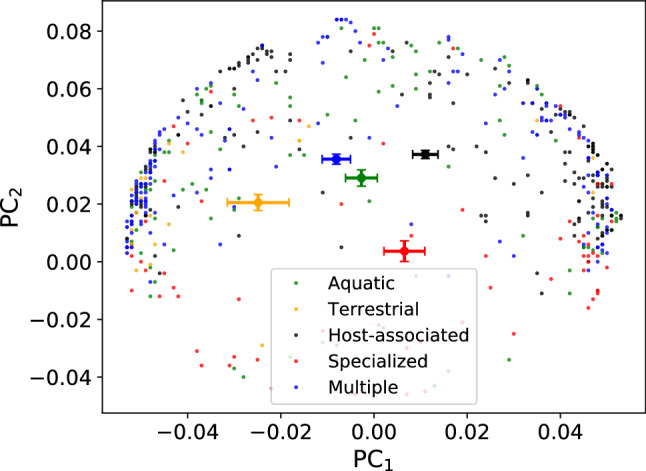
PCA using $\overline{\text{ RSCU }}$ vectors of all the species in the dataset. We projected in the PC$_1$–PC$_2$ plane the organisms with different colors, according to the habitat they live in. Note that the centroids are well separated

We analyzed the patterns of synonymous codon usage among the organisms in our dataset using the PCA on the $\overline{\text{ RSCU }}$ vectors measured for each species (see Fig. 3). The two first principal components (PC$_1$ and PC$_2$) turned out to represent as much as 71$\%$ of the total variance of $\overline{\text{ RSCU }}$ values over the 615 genomes. Interestingly, the distributions of representative points related to different phenotypic characteristics had well separated centroids in this reduced space (four panels of Fig. 3). Then, we characterised the species according to their habitat. The distributions of the organisms in the PCA plane also exhibited distinct centroids for every habitat (see Fig. 4). What we have found indicates that organisms with a specific phenotypic characteristic and living in similar environmental conditions have a similar CUB, as measured by $\overline{\text{ RSCU }}$ vectors. In other words, if a set of genomes are physically and functionally put in a relationship by the environment they thrive in, then their genes share common codon bias features.

**Fig. 5:**
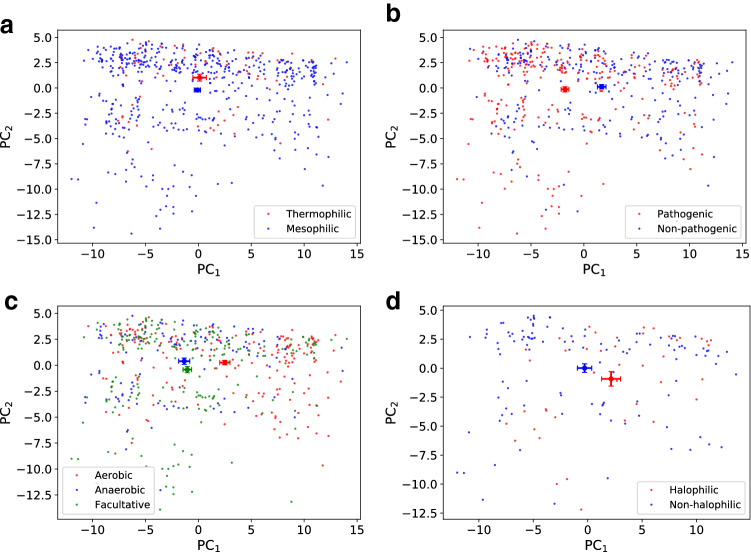
PCA using tGCN values of all the species in the dataset. The first principal component (PC$_1$) accounted for 24.7% of the total variation and the second principal component (PC$_2$) accounted for 12.6% of the total variation. We projected in the PC$_1$–PC$_2$ plane the organisms according to their phenotypic traits: temperature range (**a**), pathogenicity (**b**), oxygen requirement (**c**), salinity (**d**). Centroids were calculated as mean value with relative error bars as standard deviation of the mean. Note that the distributions of representative points have well separated centroids

**Fig. 6:**
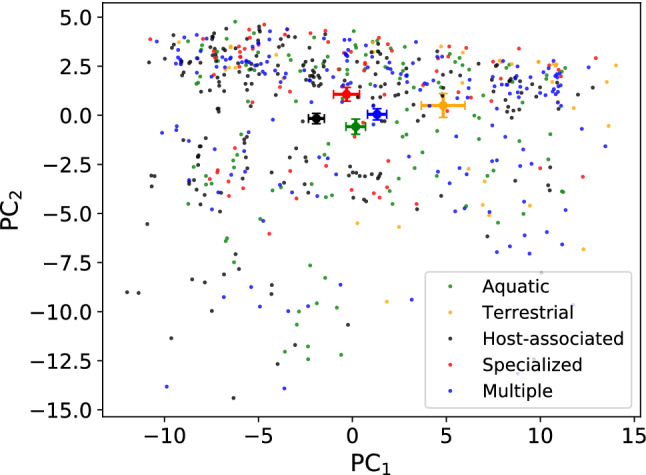
PCA using tGCN values of all the organisms in the dataset. We projected in the PC$_1$–PC$_2$ plane the organisms according to the habitat where they live. Note that, also in their case, the centroids are well separated

We then found that the different groups of organisms were also clustered, according to their lifestyles and habitats, in the space of vectors that measure the spectrum of tRNA availability to each species. The 61 components of these vectors are proportional to the number of copies of the different tRNA genes (tGCN) present in the genome of each species. The fraction of the total variance accounted for by the first two principal components in this PCA was only 37%. As we did above, we divided the organisms into groups according to their lifestyles (Fig. 5) and according to their habitats (Fig. 6). Also in this case, the centroids of the distributions are well separated in the reduced space of the first two principal components. This observation indicates that microbial species that share lifestyle and habitat do share not only signatures of a common codon usage but also co-evolved to have similar spectra of tRNA availability.

Although the centroids in the previous PCAs are visually well separated, there is a considerable spread in the data and consistent overlap between clusters of species with different lifestyles and habitats might well be present. Since this was an exploratory investigation aimed at finding just robust signals, we decided not to calculate any metric to quantify cluster separation and to assess the statistical significance of cluster separation in the PCA plane. Currently, there are no widely adopted practices used to quantify and report cluster separation in PCA scores plots, or to determine whether or not the cluster separation is statistically significant (Goodpaster and Kennedy 2011). We believe to have detected signs of an interesting correlation between lifestyles, habitats and the co-evolution of codon bias and tRNA availability of microbial species. Further assessment of the statistical validity of taking separated centroids as representative of ecological clustering in a PCA setting requires additional work (possibly using recent and promising methods based on the evaluation of the local intrinsic dimension of the data (Allegra et al. 2020)).

### tAI values

**Fig. 7:**
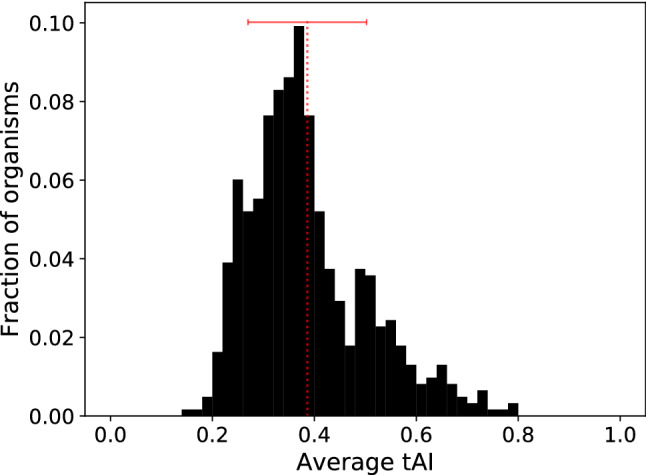
Distribution of $\overline{\text{ tAI }}$ values among the 615 species in dataset. Red dotted line denotes mean of the distribution and the error bar the standard deviation. Mean value = 0.39 and standard deviation = 0.12

In this section, we search for statistically significant differences in the distributions of tAI, considered as an index related to the efficiency of translation. We annotated each genome with its $\overline{\text{ tAI }}$ and compared the distributions of the $\overline{\text{ tAI }}$ values of different groups of organisms: thermophilic versus mesophilic; pathogenic versus non-pathogenic; halophilic versus non-halophilic; aerobic, anaerobic and facultative. We also compared the tAI signal in organisms able to live only in special environments and in those capable to deal with multiple habitats. As shown in Fig. 7, there is a wide distribution of average tAI values across the genomes, ranging between 0.15 and 0.79, with a mean value of 0.39, median of 0.36 and standard deviation of 0.12.

**Fig. 8:**
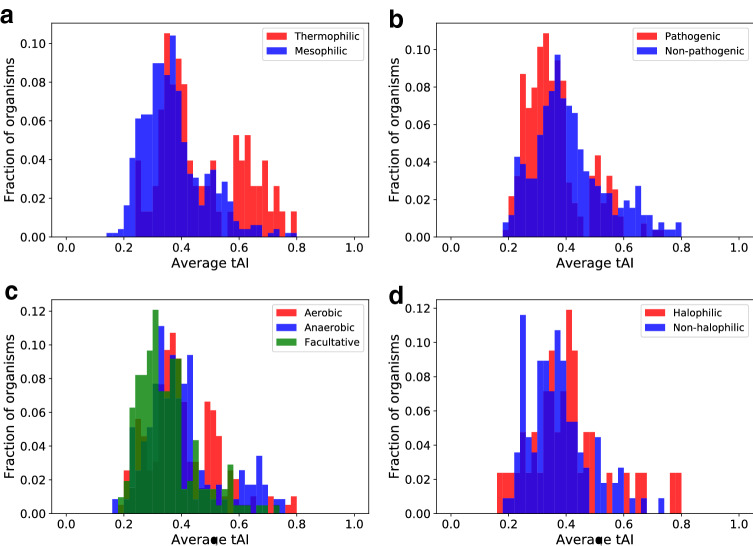
Relative frequency distribution of $\overline{\text{ tAI }}$ values of the organisms in dataset classified according to: temperature range (**a**), pathogenicity (**b**), oxygen requirement (**c**), salinity (**d**)

We examined the distribution of $\overline{\text{ tAI }}$ values among thermophilic versus mesophilic species (see Fig. 8). Organisms that live in different temperature ranges showed statistically significant differences in their $\overline{\text{ tAI }}$ values ($p = 1.62 \times 10^{-8}$, two-sided Mann–Whitney test): thermophiles demonstrated statistically significantly higher $\overline{\text{ tAI }}$ values than mesophiles. As shown in Fig. 8, the distribution among pathogenic bacteria is biased to the left compared to non-pathogenic bacteria, with pathogenic species having statistically significant lower $\overline{\text{ tAI }}$ values ($p = 8.67\times 10^{-8}$ by two-sided Mann–Whitney test). Groups of microbes classified by their oxygen requirement (Fig. 8) differed statistically significantly in the distributions of $\overline{\text{ tAI }}$ values ($p=7.09\times 10^{-8}$ by Kruskal–Wallis test). Interestingly, facultative organisms exhibited the lowest extent of translational efficiency and their distribution was statistically different from the distributions belonging to the other two groups ($p=4.53\times 10^{-8}$, two-sided Mann–Whitney test between facultative and aerobic; $p=1.35\times 10^{-4}$ between facultative and anaerobic). The difference between aerobic and anaerobic species was not statistically significant ($p=0.301$ by two-sided Mann–Whitney test). Groups of microbes that live in environments which differ in their salinity levels (Fig. 8) did not demonstrate statistically significant differences among them ($p=0.161$ by two-sided Mann–Whitney test).

**Fig. 9:**
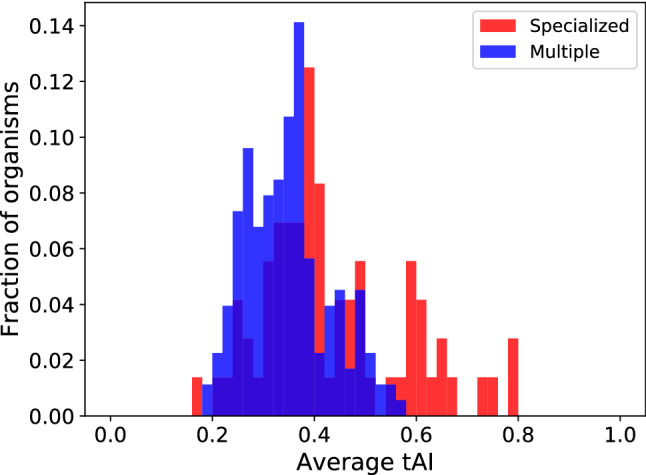
Relative frequency distribution of $\overline{\text{ tAI }}$ values of microbes divided into two groups: organisms that live in a specialised environment and organisms that live in multiple habitats

We then turned to analyze the differences between organisms living in different habitat conditions (see Fig. 9). Intriguingly, we found that organisms living in multiple habitats have statistically significant lower $\overline{\text{ tAI }}$ values than organisms living in specialised habitats ($p=2.66\times 10^{-6}$, two-sided Mann–Whitney test). This result is consistent with the results presented above for the other phenotypic traits and generalises them. Pathogenic bacteria often live in multiple environments outside and within their host, and facultative organisms live in environments with and without oxygen. On the other hand, thermophiles (found above to have a higher extent of translational efficiency) are usually restricted to a specific environment with a specific temperature.

## Discussion

This work had, as a starting point, the idea that the speed at which codons are decoded on the ribosomes is regulated, given the codon bias, by the cellular concentration of the tRNAs that recognise them. Moreover, we had also in mind to find, in bacteria and archaea, correlations between codon bias, inferred tRNA availability, and their adaptation to the environments they thrive in. In general, the analysis of CUB has been widely used to characterise both specific and general properties of genes from communities of microorganisms (Ran and Higgs 2012). Many evidences and signals from the literature suggest that CUB can affect cellular fitness and might be associated with the lifestyle of the organism. To explore this hypothesis, we studied here the relationship between CUB and the ecology of 544 bacterial and 71 archaeal species. The first results worth to be mentioned refer to the distribution of RSCUs (Figs. 1, 2). Each gene in a genome was tagged by a 61-dimensional RSCU vector and the codon bias fingerprint of each species was expressed as the average $\overline{\text{ RSCU }}$ vector. Each genome has its own, robust, codon bias signature; the internal fluctuations of the RSCUs of each gene in a genome are smaller than the fluctuations of $\overline{\text{ RSCU }}$s of the different species. Moreover, PCA of the space of $\overline{\text{ RSCU }}$s confirmed that the different phenotypic traits of the species are reflected in their codon preferences. *Species sharing similar adaptation to the environment also share similar CUB*. In many genomes, as reminded in the Introduction, the most used codons are those matching the most abundant tRNAs, suggesting that selection for optimal translation could be the evolutionary pressure driving both, CUB and tRNA availability. This perspective is nowadays experimentally investigated, mainly by ribosomal profiling techniques. We believe that a systematic comparison between computational predictions and *in vivo* measured rates of proteins synthesis in several microbial species will soon be possible, opening a perspective in which modeling and empirical observation will eventually cooperate toward a better understanding of gene expression levels and their regulation. Overall, our findings can be summarised as follows: i) PCA showed that species sharing specific phenotypic characteristics and living in similar environments have both similar codon preferences, as statistically represented by average RSCUs, and similar tRNA availability, as estimated by the tGCN; ii) the average tAI of each genome, that we roughly associate with translational efficiency (as measured by average rate of protein synthesis), is lower in organisms able to live in a wide range of habitats; iii) pathogenic species also have a lower average tAI than non-pathogenic ones, consistently with the view that adaptability to multiple environments is a characteristic trait of pathogens; iv) facultative organisms, which are able to grow in the presence or in the absence of oxygen, have also lower values of average tAI than more specialised aerobic/anaerobic species; and v) mesophile species also tend to have a lower average tAI than extremophile species. In a nutshell, these results clearly show that the co-evolution of codon bias and tRNA availability is strictly associated with the adaptation of Bacteria and Archaea to their environments, with an apparent sharpening of the selection in the species that face harsher environments, basically an expected result.

Let us now try a critical assessment of what we have observed. Of course, the idea of associating ecological adaptation and codon adaptation is not new (Botzman and Margalit 2011; Jiang et al. 2008). To elaborate on this idea, we added the information about the adaptation of codon usage to the genomic tRNA gene pool (Reis et al. 2004; Sharp and Li 1987) where translational selection is known to be present. It has been observed in several species that in vivo concentration of a tRNA bearing a certain anticodon correlates with the number of gene copies coding for this tRNA (for example, paradigmatically, in *S. cerevisiae*, Pearson’s $r = 0.91$ (Percudani et al. 1997)). This correlation is widely used to infer the expected in vivo average tRNA pools from genome sequence-only information, though, depending on the context, it should be assumed with awareness of its limitations. To have a robust correlation among actual tRNA levels, codon bias and levels of protein expression one should await, as mentioned above, further refinements and broad diffusion of computational and experimental techniques.

However, using the tGCNs, we carried out a PCA of the tRNA repertoire belonging to the species considered in this study. This analysis was less convincing than the one based on $\overline{\text{ RSCU }}$s (the first two principal components explained, in this latter case, only 37% of the total variance), but roughly confirmed that differences in the lifestyle can be associated with different patterns of tGCN (number of tRNA gene copies) in the species we considered.

On the use of tAI as a proxy of translational efficiency of single genes, as we did (see also the tAI section in Materials and Methods), several lines of evidence indicate that the tAI-based translation efficiency values are biologically significant. Very often to higher tAI, do correspond higher protein abundances (Reis et al. 2004; Man and Pilpel 2007; Tuller et al. 2007). Note also that protein expression levels of single genes can be customarily increased, as in biotechnological applications, by tailoring induced mutations that increase codon–tRNA adaptation (Percudani et al. 1997; Tuller et al. 2007). That points to a causal (robust) relationship between codon usage and expression levels, mediated by cellular availability of tRNAs.

Moreover, to have a quantitative evaluation of the relative ”optimality” in the adaptation of different organisms to their environments we have simply used $\overline{\text{ tAI }}$, the average of the tAI values of all CDSs (coding regions, genes) in a genome. Our analysis revealed a large variability of this statistic over the different species: there are organisms showing very high degrees of translation efficiency and organisms exhibiting very low $\overline{\text{ tAI }}$ values. The findings of Botzman and Margalit (Botzman and Margalit 2011) motivated us to compare the distributions of $\overline{\text{ tAI }}$ values in groups of species with different phenotypic characteristics. Remarkably, we found a correlation between the extent of translational efficiency and the lifestyle of the organism. Even more interestingly, we found that these differences are related to whether the organism can thrive in multiple habitats or just in a single, peculiar, habitat. We observe that organisms living in a demanding specialised habitat (e.g. hyperthermophiles) have higher translational adaptation (as measured by higher $\overline{\text{ tAI }}$ values); whereas, species that live in multiple environments display lower $\overline{\text{ tAI }}$ values, suggesting that in more adaptable species, the co-evolution of codon bias and tRNA pools is under a lower selective pressure.

With this work, we believe to have contributed to show that there is an evolutionary convergence of codon bias and tRNA availability in groups of organisms sharing similar physiology and living in similar habitats. The understanding of the overall picture is still far from complete, nevertheless let us make here some points about the plausibility of our observations and some general conclusive remarks. The adaptation process that is shared by organisms living in similar environments might include several factors, still to be properly investigated and understood: (i) successful exchange of genes by lateral transfer, as a source of adaptation; (ii) possibly universal mechanisms for the convergence toward shared values of an external environmental parameter such as the optimal growth temperature or pressure or salinity (as, e.g. in thermophile, barophiles and halophiles); (iii) common mechanisms of adaptation to abundance/lack of nutrients that could sculpt the relative usage of specific codons in the genomes; and (iv) in pathogens successfully adapted to their hosts, common traits of genetic variability may emerge, affect codon usage and pave the way to escape the host-immune system (Carbone et al. 2005).

Several observations support the set of considerations given above. Referring to the possible role of gene transfer consider, for instance, the case of bacterium *Aquifex aeolicus* which occupies the hyperthermophilic niche otherwise dominated by Archaea. Genome analysis, suggest that the archaeal genes in *Aquifex* have been introduced by horizontal gene transfer, on top of a typical bacterial gene repertoire, and have been retained owing to the specific selective advantage they provided by enabling the bacterium to thrive in high-temperature habitat (Aravind et al. 1998). A similar gene transfer has been observed for another hyperthermophilic bacterium, *Thermotoga maritima* (Carbone et al. 2005). Furthermore, communities of microbes have been shown to share similar tRNA pools to facilitate horizontal gene transfer (Tuller et al. 2011), which also implies a limited choice of preferred codons that are cognate to the shared tRNA pools. This is consistent with the findings of the present work.

Freilich et al. showed that most bacterial organisms choose one of the two alternative ecological strategies: either living in multiple habitats with a large extent of co-habitation, associated with a typically fast rate of growth, or living in a specialised niche with little co-habitation, associated with a typically slow rate of growth (Freilich et al. 2009). Independently, Rocha demonstrated that fast growing bacteria have more tRNA genes of fewer types and suggested that the translation in those organisms depends on fast tRNA diffusion to the ribosome (Rocha 2004; Vieira-Silva and Rocha 2010). Our findings tie these two results together and suggest that organisms may adjust to metabolic variability and competition by maintaining a low extent of adaptation of their genes to the tRNA pool (reflected by their low $\overline{\text{ tAI }}$ values).

To our knowledge this is the first large-scale study that examines, though indirectly, the role of translational efficiency in the adaptation of Bacteria and Archaea to the environment they live in. Along ribosomal translation elongation, synonymous codons are decoded at different rates, reasonably due to tRNA availability and to the availability and synthetic cost of different amino acids. The fact that each species has its own codon bias suggests that, in perspective, one should interpret the codon bias that emerges from the evolution of a species as the result of multiple trade-offs between translation efficiency, biosynthetic costs, and availability of nutrients typical of different environments. From the phylogenetic point of view, this adds additional source of information that deserves further systematic investigation.

## Supplementary Information

Below is the link to the electronic supplementary material.

Supplementary material 1 (xlsx 762 KB)

## Data Availability

All data are available upon request.

## Declarations

### Code availability

All code is available upon request.
